# Cytotoxic effects of hydroalcoholic extract of *Cuscuta chinensis* on PC3 and MCF7 cancer cell lines 

**Published:** 2021

**Authors:** Fatemeh Karimi Dermani, Massoud Saidijam, Rezvan Najafi, Shirin Moradkhani, Zahra Mohammadzaheri, Negar Beiranvand, Samane Mohammadi, Noushin Shabab, Ramazan Kalvandi, Fatemeh Zeraati

**Affiliations:** 1 *Research Center for Molecular Medicine, Hamadan University of Medical Sciences, Hamadan, Iran*; 2 *Medicinal Plants and Natural Products Research Center, Hamadan University of Medical Sciences, Hamadan, Iran*; 3 *Department of pharmacognosy, School of Pharmacy, Hamadan University of Medical Sciences, Hamadan, Iran*; 4 *Natural Resources Department, Hamadan Agricultural and Natural Resources Research and Education Center, AREEO, Hamadan, Iran*; 5 *Department of Pharmacology and Toxicology, School of Pharmacy, Hamadan University of Medical Sciences, Hamadan, Iran*

**Keywords:** Cuscuta. Chinensis, Chemoprevention, Prostate cancer, Breast cancer, Apoptosis

## Abstract

**Objective:**

Chemoprevention of cancer by application of natural phytochemical compounds has been used to prevent, delay or suppress cancer progression. *Cuscuta chinensis* a traditional Iranian medicinal herb, has biological properties including anticancer, anti-aging, immuno-stimulatory and antioxidant effects. In this study, anti-proliferative effects of hydroalcoholic extract of *C. chinensis* on prostate (PC3) and breast (MCF7) cancer cell lines were investigated.

**Materials and Methods:**

In the current study, we investigated treatment of PC3 cells with different concentrations of *C. chinensis* (0, 100, 200, 300, 400, and 500 µg/ml) for 24 and 48 hr; also, MCF7 cells were treated with various concentrations (0-600 µg/ml) of *C. chinensis* for 48 and 72 hr and cell viability was assessed by 3-(4, 5-dimethylthiazol-2-yl)-2, 5-diphenyltetrazolium bromide (MTT) assay. mRNA expression of BCL2 Associated X (*Bax*), B-cell lymphoma 2 (*Bcl2*), Cysteine-aspartic proteases (*Caspase3*) and Phosphatase and tensin homolog (*PTEN*) were analyzed by quantitative real-time PCR. Annexin V/PI staining and lactate dehydrogenase (LDH) cytotoxicity assay were used to detect apoptosis.

**Results:**

*C. chinensis* decreased PC3 and MCF7 cells viability in a dose- and time-dependent manner (p<0.01 to p<0.001). The gene expression of *BAX/Bcl2* ratio, *Caspase3 *and *PTEN* increased in *C. chinensis*-treated cells compared to the control group. *C. chinensis* induced apoptosis (p<0.001) and LDH activity (p<0.01 to p<0.001).

**Conclusion:**

Our findings suggest that *C. chinensis* extract is able to inhibit proliferation and induce apoptosis in PC3 and MCF7 cell lines. Therefore, *C. chinensis* extract exerts antitumor activity against cancer cells.

## Introduction

Cancer is one of the major burden disease and prostate cancer is the most common leading cause of death in men after lung cancer throughout the world (Jemal et al., 2009 ▶). Studies displayed that anticancer drugs are usually known to cause severe adverse effects and many complications such as suppression of the immune system, which have limited their use (Ghazanfari et al., 2013 ▶). Several factors make prostate cancer an ideal target disease for chemoprevention such as long latency, high incidence, and tumor markers availability (prostate-specific antigen, PSA), identifiable preneoplastic lesions; also, it is a very heterogeneous disease with a large subgroup of patients with nonaggressive disease (Van Poppel and Tombal, 2011 ▶).

Breast cancer is the most prevalent cancer in women worldwide in terms of incidence and mortality (Tryggvadóttir et al., 2010 ▶). Metastasis of breast cancer cells to the bone, lung and other vital organs is responsible for most cancer deaths (Kang, 2009 ▶). Common treatments for breast cancer, combination of surgery, radiation therapy, chemotherapy, hormone therapy and/or targeted therapy, are less than satisfactory due to high rates of metastasis (Redig and McAllister, 2013 ▶). 

 Recent studies demonstrated that medicinal plants act as anticancer agents which have antiproliferative effects on breast cancer cell lines (Lewandowska et al., 2014 ▶; Behzad et al., 2014 ▶) .

Chemoprevention is defined as the use of specific natural (dietary) or synthetic agents to prevent, delay, or slow down the development and progression of cancer (Van Poppel and Tombal, 2011 ▶).

The use of herbal medicine as an alternative treatment has been embraced throughout the world. Herbal medicines as rich sources of natural anticancer materials, are potential candidates for development of anticancer drugs. *Cuscuta* (dodder) is one of the medicinal herbs belonging to the Convolvulaceae plant family which is distributed in tropical and temperate regions, and possesses many species (Jafarian et al., 2014). *C. chinensis *Lam known as aftimun, is commonly used in Indian traditional herbal medicines with many therapeutic effects. In clinical settings, *C. chinensis* has been considered to improve sexual function, prevent aging, and regulate the immune system (Zheng et al., 1998 ▶). Previous studies showed that *Cuscuta* possess a number of biological properties including anticancer (Nisa et al., 1986 ▶; Umehara et al., 2004 ▶), antiaging (Jian-Hui et al., 2003 ▶), and immuno-stimulatory and antioxidant effects (Bao et al., 2002 ▶). It was also used to improve the liver, kidney and vision complications (Yenet al., 2007 ▶).

According to our knowledge, there have been few reports on the effect of *C. chinensis* on various cancer cell lines; particularly, there has been no report on MCF7 and PC3 cell lines. The aim of this study was to evaluate the cytotoxic mechanism of *C. chinensis*in PC3 and MCF7 lines. 

## Materials and Methods

**Preparation of plant extract**

*C. chinensis *was obtained from the cultivated populations in botanical garden in Hamedan province. The plant material was authenticated in Department of Pharmacognosy, School of Pharmacy, Hamadan University of Medical Sciences, Hamadan, Iran by Dr. Ramazan Kalvandi and a voucher specimen (No. 444) was deposited. Dried plant material (100 g) was powdered, macerated in hydroalcohol (80%) for three times, in three days. Then, the resulting filtrate was dried using a rotary evaporator. The resulting extract was kept in a sterile vial in a dark and cool place for future investigations (Amidi et al., 2016 ▶)

**Reagents**

RPMI-1640, DMEM (Dulbecco’s modified Eagle’s medium), fetal bovine serum (FBS) and penicillin/streptomycin were obtained from Gibco, Invitrogen. MTT [3-(4, 5 Dimethylthiazol-2-yl)-2, 5-diphenyltetraolium bromide] powder and phosphate buffered saline (PBS) were obtained from Merck (Germany). Prostate cancer cell line (PC3) and breast cancer cell line (MCF7) were purchased from National Cell Bank of Iran, Pastor Institute, Iran.Dimethyl Sulfoxide (DMSO) was from Sigma-Aldrich. RNX- Plus solutionwas procured from Sinaclone, Iran. cDNA synthesis kit was obtained from Thermo Scientific. SYBR Premix Ex Taq II Kits were purchased from Takara, USA. LDH-Cytotoxicity Assay Kit ΙΙ was obtained from Abcam, USA.

**Cell culture**

The human prostate cancer cell line PC3 and breast cancer cell line MCF7 were grown in RPMI and DMEM medium containing 10% fetal bovine serum (FBS) and 100 U/ml penicillin and 100 g/ml streptomycin (P/S) in humidified air (5% CO_2_ atmosphere) at 37˚C, respectively (Dermani etal., 2018) 

**Cell viability assay**

The inhibitory effects of* C. chinensis* on cell growth were determined by the analysis of viable cells using the 3-(4, 5-dimethylthiazol-2-yl)-2, 5-diphenyltetrazolium bromide (MTT) assay (Dermani et al., 2018). MTT reduction assay is used for assessing cells viability; this is a colorimetric assay based on metabolic reduction of soluble MTT by mitochondrial enzyme activity of viable tumor cells, into an insoluble purple formazan compound product. PC3 and MCF7 cells were seeded in 96-well plates at a density of 7000 cells per well and then PC3 cells were treated with different concentrations of *C. chinensis *(0, 100, 200, 300, 400, and 500 µg/ml) for 24 and 48 hr; MCF7 cells were treated with (0, 100, 200, 300, 400, 500, and 600 µg/ml) of *C. chinensis* 48 and 72 hr. After the incubation time, 10 µl of MTT solution was added to each well and the mixture was further incubated for 4hr at 37°C. Cell culture medium in the wells was removed and then 100μl of DMSO was added to dissolve the formazan compound. The optical density (OD) was read by an automatic microplate reader at 570 nm (Rayto RT–2100c, China). 

**Real-time PCR**

To evaluate the alterations in the gene expression patterns, PC3 and MCF7 cells were seeded in 6-well plates at a density of 5×10^5^ cells per well and then PC3 cells were treated with different concentrations of *C. chinensis *(0, 200, 300, and 400 µg/ml) for 24 and 48 hr and MCF7 cells were treated with (0, 100, 200, and 300 µg/ml) of *C. chinensis* for 48 and 72 hr. Then, total RNA was isolated from the treated and untreated cells using the RNX- Plus solution according to the manufacturer’s protocol. RNA concentration and purity were evaluated by optical density measurement using a Nano-Drop spectrophotometer. The cDNA was synthetized using a kit (Thermo Scientific) according to the manufacturer's protocol by incubation for 5 min at 25°C, 1 hr at 42°C and 5 min at 70°C. Total volume of reaction mixture for real time PCR was 20 μl and contained 1 μl cDNA, 7 μl H_2_O, 10 μl SYBR Premix Ex Taq II kits (Takara), and 1 μl of 10 pmol/μl specific primers (*Bax*, *Bcl2*, *Caspase3* and *PTEN*) (Table 1).

 Thermal cycling for mixture was programmed as follows: an initial denaturation at 95°C for 10 min, 40 cycles of denaturing at 95°C for 15 sec, annealing at 60°C for 30 sec and extension for 30 sec at 72°C. The PCR was run on a light cycler real-time PCR system in triplicate. The levels of RNA were normalized to *18s rRNA*. Relative gene expression was reported by 2^−∆∆CT^ method analysis (Livak and Schmittgen, 2001 ▶). 

**Table 1:** Primers used for real-time PCR

**Gene**	**Sense strand**	**Antisense strand**
*PTEN*	CCCACGAAGCCTTGTTTACC	AGCTGTTGTAAGAGGTGCCCTGGAA
*Caspase*	TTGTCGGCATACTGTTTC	CAGCACCTGGTTATTATTCT
*BAX*	CGCCGTGGACACAGACTC	GCCTTGAGCACCAGTTTG
*Bcl-2*	TGGAGAGTGCTGAAGATTGA	GTCTACTTCCTCTGTGATGTTGTA
*18srRNA*	GTAACCCGTTGAACCCCATT	CCATCCAATCGGTAGTAGCG

**LDH cytotoxicity assay**

Analyzing cell growth inhibition and/or cell death has been an important component of many biological researches, especially in cancer treatment development. LDH activity was evaluated by LDH-Cytotoxicity Assay Kit ΙΙ (Abcam, USA). Briefly, a suspension of cells (5×10^5^) was prepared in several groups as negative control (culture medium with no cells), positive control (cell lysis solution) and test sample including PC3 and MCF7 cells cultured in 96 well plates at a density of 7000 cells per well. PC3 cells were treated with different concentrations of *C. chinensis *(0, 200, 300, and 400 µg/ml) and incubated for 24 and 48 hr and MCF7 cells were treated with (0, 100, 200, and 300 µg/ml) of *C. chinensis* and incubated for 48 and 72 hr. Cells were centrifuged at 600 g for 10 min to precipitate the cells. After that, the clear medium solution (10 μl/well) was transferred into an optically clear 96-well plate. Then, 100 μl LDH Reaction Mix was added to each well, mixed and incubated for 30 min at room temperature. The assay carried out as described by the manufacturer. Absorbance was measured by an enzyme linked immunosorbent assay (ELISA) micro titer plate reader at 450 nm (Smith et al., 2011 ▶). The assay was conducted in triplicate.

**Apoptosis assay**

Apoptosis assay in PC3 and MCF7 cells were performed using Annexin V-FITC/PI assay. Cells were seeded in 6-well plates at a density of 5×10^5^ cells/well and PC3 cells treated with different concentrations of *C. chinensis *(0, 200, 300, and 400 µg/ml) 24 and 48 hr and MCF7 cells were treated with (0, 100, 200, and 300 µg/ml) *C. chinensis* for 48 and 72 hr; after that, cells were trypsinized and centrifuged at 1500 g for 5 min, then the cells were stained with annexin V and PI at room temperature at night. Annexin V-FITC was used to detect the phosphatidylserine during apoptosis. To distinguish necrotic cells, Propidium Iodide (PI) staining was employed. The cells were evaluated by a FACS caliber cytometer (Becton Dickinson, San Diego, CA) and Cell Quest software (Dermani et al., 2017). The assay was conducted in duplicate.

**Statistical analysis**

Data were analyzed and are presented as the mean±standard deviation. The differences among groups were assessed by one-way ANOVA and Tukey’s *post hoc* tests. A p<0.05 was perceived as statistically significant.

## Results

**Effect of** ***C. chinensis*** **on cell viability**

**Figure 1:**
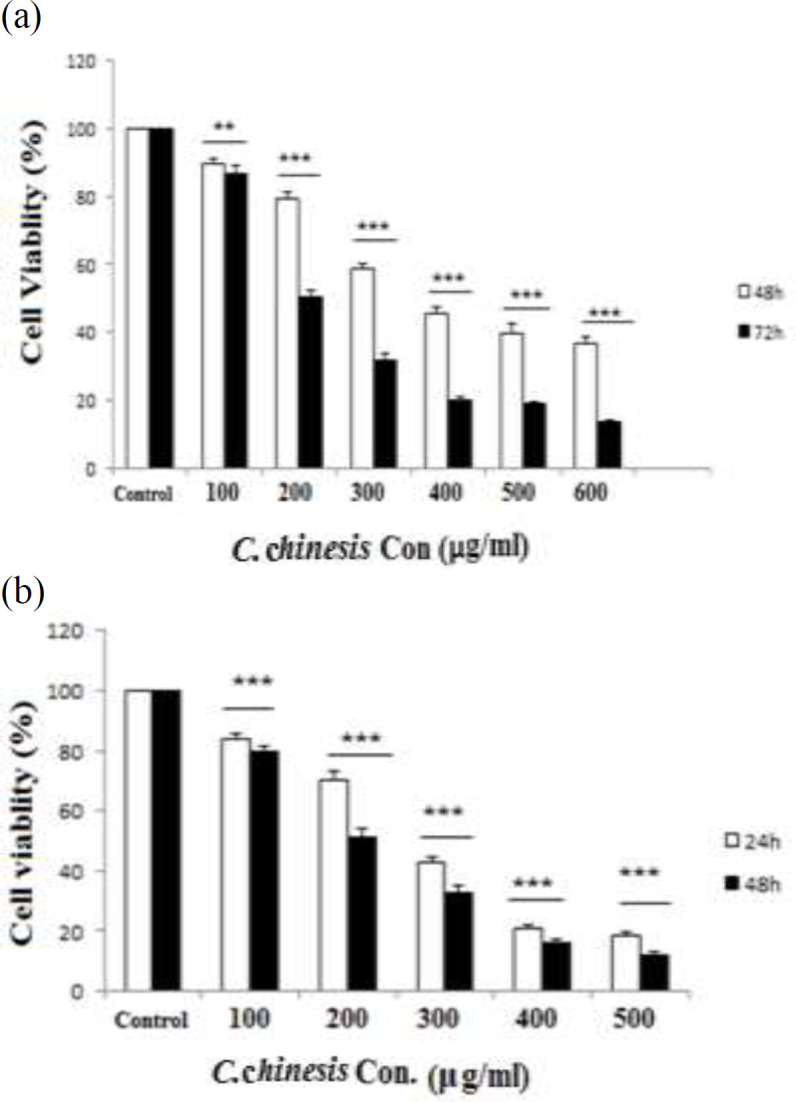
Effect of *C. chinensis *on MCF7 (a) and PC3 (b) cells viability. PC3 cells were treated with 0, 200, 300, and 400 µg/ml of *C. chinensis *and for 24 and 48 hr and MCF7 cells were treated with 0, 100, 200, and 300 µg/ml of *C. chinensis* for 48 and 72 hr and their viability were examined by MTT assay. Data are reported as the mean±SEM (n=8). **p<0.01 and ***p<0.001 compared to the control

We investigated the effect of various concentrations of *C. chinensis* on PC3 and MCF7 cells viability by MTT assay. As shown in Figure 1, *C. chinensis* decreased the percentage of viable cells compared with untreated cells in a dose- and time-dependent manner (p<0.01 for the effect of 100 µg/ml on MCF7 cells and p<0.001). 

The 50% inhibition concentration (IC50) values were determined as 400 and 200 μg/ml after 48 and 72 hr for MCF7 (Figure 1a) and 300 and 200 μg/ml after 24 and 48 hr, for PC3 (Figure 1b), respectively.

**Effect of** ***C. chinensis*** **on expression of** ***Bax*** **,** ***Bcl2*** **,** ***Caspase3*** **, and** ***PTEN***

**Figure 2:**
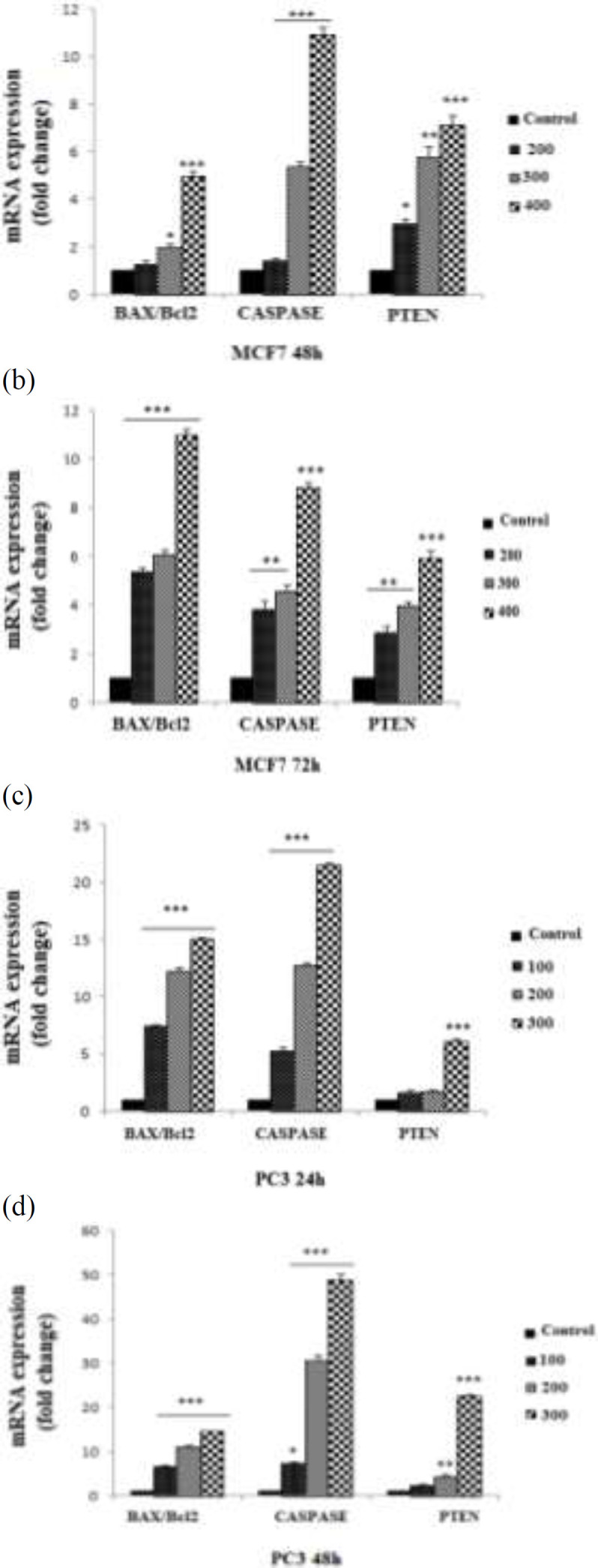
Effect of *C. chinensis* on *BAX/Bcl2*, *CASPASE3* and *PTEN* expression. Relative expression genes in MCF7 (a and b) and PC3 (c and d) cells was determined by real time PCR. PC3 cells were treated with 0, 200, 300, and 400 µg/ml of *C. chinensis *for 24 and 48 hr and MCF7 cells were treated with 0, 100, 200, and 300 µg/ml of *C. chinensis* for 48 and 72 hr. The 2^−ΔΔCT^ method was used for data analysis. Data are reported as the mean±SEM. *p<0.05, **p<0.01, and ***p<0.001 show significant differences compared to the control

The level of apoptosis-related genes (*Bax*, *Bcl2*, *Caspase3* and *PTEN*) with or without *C. chinensis* treatment, was assessed using quantitative real-time PCR. As shown in Figure 2, the expression of *Bax/Bcl2* ratio (p<0.05 to p<0.001), *Caspase3* and *PTEN* genes (p<0.05 to p<0.001) significantly increased by treatment of *C. chinensis* in PC3 and MCF7 cells.

**LDH test results**

To detect the effect of *C. chinensis* on LDH released from damaged cells into the culture medium, lactate dehydrogenase assay was performed. The percentage of viability of prostate cancer cells decreased significantly when treated with different concentration of *C. chinensis* and the level of enzyme activity measured in lactate dehydrogenase assay progressed in a dose- and time-dependent manner due to the release of enzyme caused by irreversible cell death. 

**Figure 3:**
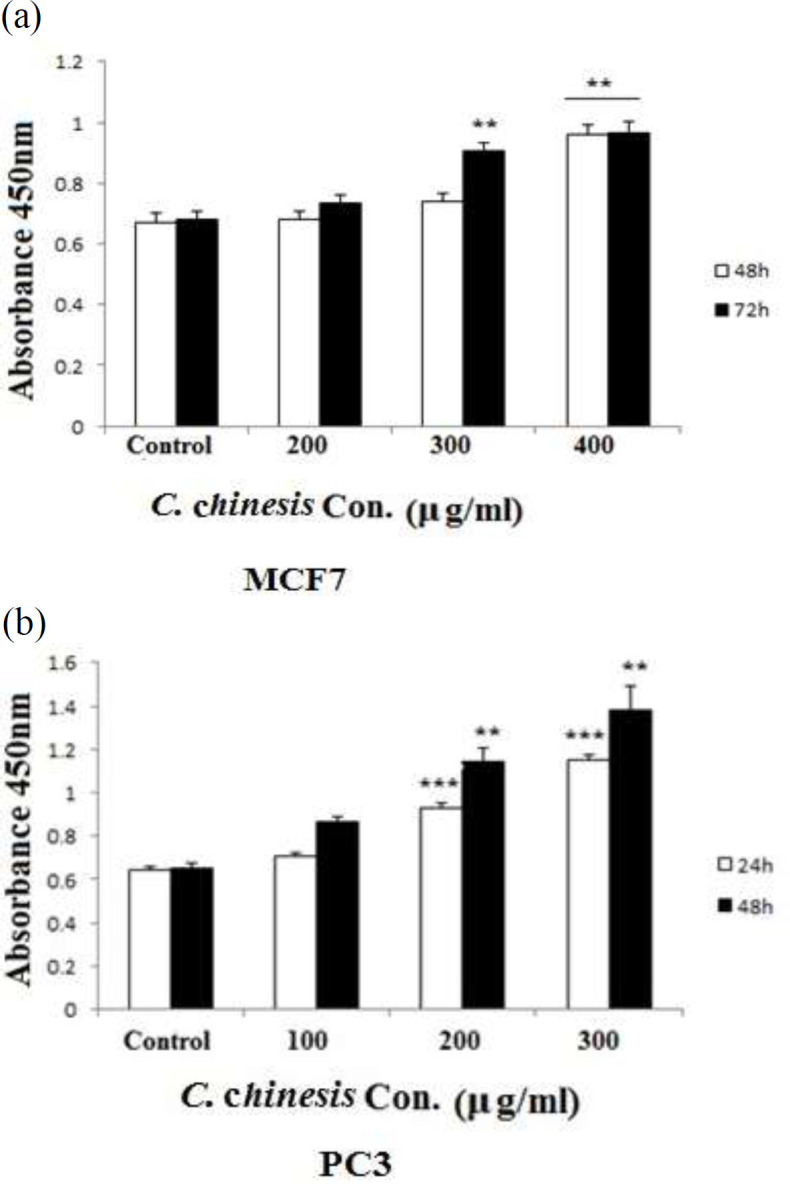
Effect of *C. chinensis *on MCF7 (a) and PC3 (b) on LDH activity. PC3 cells were treated with 0, 200, 300, and 400 µg/ml of *C. chinensis *for 24 and 48 hr and MCF7 cells were treated with 0, 100, 200, and 300 µg/ml of *C. chinensis* for 48 and 72 hr. LDH activity was assessed in cell lysate using colorimetric kit. *C.chinensis* increased LDH activity. Data are expressed as the mean±SEM. **p<0.01 and ***p<0.001 show significant differences versus the control. LDH: Lactate dehydrogenase

The result of lactate dehydrogenase assay using different concentrations of *C. chinensis* is presented in Figure 3. There was a dose- and time-dependent increase in the level of lactate dehydrogenase enzyme released by damaged cells of PC3 and MCF7 cell lines when treated with *C. chinensis* (p<0.01 to p<0.001). So, *C. chinensis* may possess a cytotoxic activity. 

**Effect of** ***C. chinensis*** **on apoptosis**

**Figure 4:**
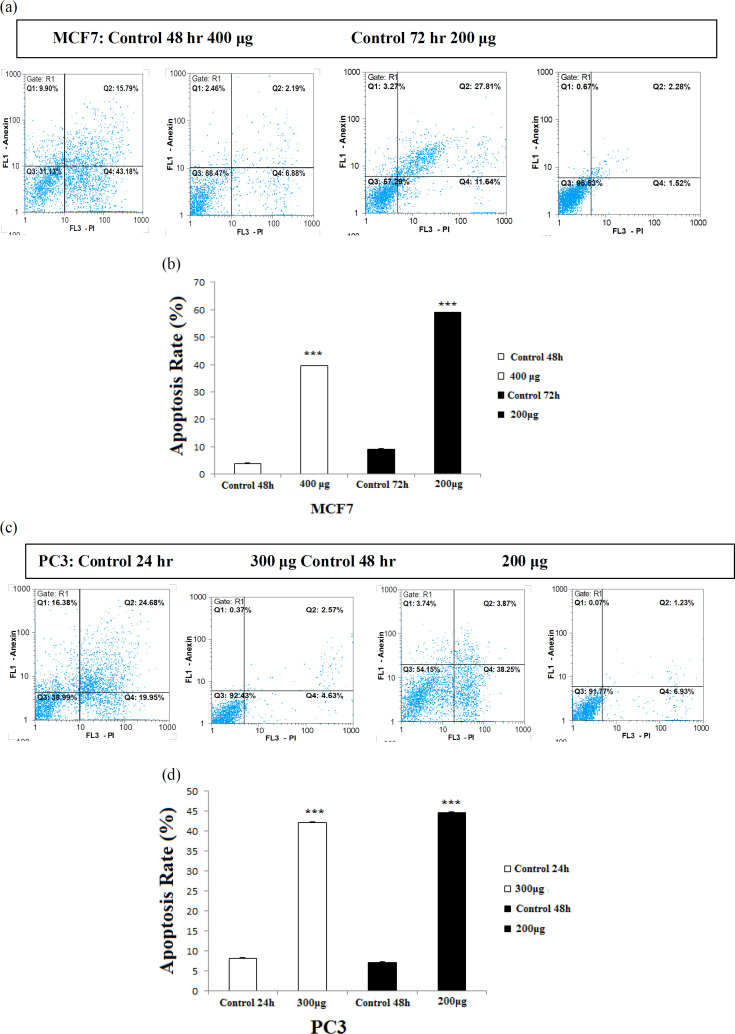
Representative flow cytometric analysis of treatment of MCF7 (a and b) for 48 and 72 hr with respectively 400 and 200 μM *C. chinensis* and PC3 (c and d) with 300 and 200 μM *C. chinensis* for respectively 24 and 48 hr. Data are expressed as mean±SEM. ***p<0.001 shows significant differences vs untreated cells

To examine the effect of *C. chinensis* on apoptosis, PC3 and MCF7 cells were stained with Annexin-V/PI. As shown in Figure 4 and Table 2, exposure of PC3 cells to 250 and 200 μM*C. chinensis*for 24 and 48 hr and treatment of MCF7 cells for 48 hr and 72 hr with respectively 400 and 200 μM *C. chinensis*, significantly increased the number of apoptotic cells in a time- and dose-dependent manner (p<0.001 for all cases). 

## Discussion

Cancer is one of the main causes of death in many countries and is one of the major problems in the present century (Torre et al., 2012 ▶). 

Over the last years, chemoprevention by naturally occurring compounds has been emerged as an appealing and cost-effective method for perturbation of cancer initiation and progression. The main benefits of natural compounds include mild side effects compared with chemically synthetized drugs (Sporn and Suh, 2000 ▶). 

In the current study, extract of *C. chinensis* was assessed for its anti-cancer properties in human cancer cell lines (PC3 and MCF7) and we investigated the effect of *C. chinensis *on cell viability, cell cycle and apoptosis. Our novel results in this study, indicated that *C. chinensis* treatment inhibited PC3 and MCF7 cells proliferation and induced apoptosis and LDH cytotoxic activity. 

Based on our findings, *C. chinensis* extract has anti-proliferative and suppressive effects on viability in PC3 and MCF7 cells. The MTT and LDH assay discovered that *C. chinensis* significantly decreased cell growth in a time- and dose-dependent manner. In the present study, the IC50 value of *C. chinensis* was determined 400 and 200 μg/ml after 48 hr and 72 hr in MCF7 and 300 and 200 μg/ml after 24 and 48 hr, in PC3 respectively. Our data suggest that PC3 cells are more sensitive to *C. chinensis* than MCF7 cells. 

These findings are in agreement with previous studies representing that *Cuscuta* extract can inhibit proliferation and induce apoptosis and cell cycle arrest in a wide variety of cancer cells (Ghazanfari et al., 2013 ▶; Jafarian et al., 2014; Zeraati et al., 2015 ▶; Sepehr et al., 2011 ▶).

**Table 2:** Effect of *C. chinensis* on percentage of apotosis in MCF7 and PC3 cell lines

	**Cell lines**			
**Apoptosis rate (%)**	**PC3 (24 hr)**	**PC3 (48 hr)**	**MCF7 (48 hr)**	**MCF7 (72 hr)**
	58.97	39.45	44.63	42.12

These suggest that *Cuscuta* is a competitive candidate as a novel anti-cancer agent. However, the studies on apoptotic activity of *Cuscuta*are limited and have remained to be uncovered. Ghazanfari et al. demonstrated that *Cuscuta* extract has antioxidant and anticancer effects on melanoma cell line (SK-MEL-3) and human Burkitt lymphoma (Raji). Their results showed that low concentrations of *Cuscuta *were needed to induce anti-tumor effects (Jafarian et al., 2014). Sepehr et al. showed that flavonoid extracts of Cuscuta seed stem and its host plant (vine) of *C. kotschyanahas *have anticancer effect mediated by suppression of proliferation and apoptosis induction in human breast cancer cell line (MCF7) (Sepehr et al., 2011 ▶). In addition, Zeraati et al. demonstrated that *C. chinensis* had cytotoxic effects against human Caucasian acute lymphoblastic leukemia (CCRF-CME) and other human lymphocyte, Jurkat (JM) cell lines (Zeraati et al., 2015 ▶). In agreement with our results, another study demonstrated that equiguard, a dietary supplement consisted of standardized extract from nine herbs comprising *C. chinensis *(ethanol extract of seed), significantly reduced cell growth and induced apoptosis in prostate carcinoma (Hsieh et al., 2002 ▶).

In contrast with our study, the crude extract of *C. chinensis* promoted proliferation and differentiation of osteoblasts from their precursor cells (Yao et al., 2005 ▶) Besides, a resin glycoside isolated from the seeds extract of *C. chinensis* (Convolvulaceae) demonstrated potency for stimulating MCF-7 and T47D human breast cancer cells proliferation at 10 μM concentration (Umehara et al., 2004 ▶).

In this study, we reported the involvement of *C. chinensis *in molecular mechanism of *Bax/Bcl2*, *caspase3* and *PTEN *induced apoptosis and suppressed proliferation in human prostate and breast cancers. PC3 and MCF7 cells treated with *C. chinensis* displayed typical apoptotic features, comprising shrinkage of cytoplasm, blebbing of the plasma membrane, condensation of nuclear chromatin, chromosomal DNA fragmentation, and cleavage of the cells into membrane-enclosed vesicles designated as apoptotic bodies (Kerr et al., 1972 ▶). 

Phosphatidylserine (PS), which is typically situated in the plasma membrane inner leaflet, at the beginning of apoptosis, is translocated and exposed to the outer wall, thus providing a “molecular marker” on apoptotic cells (Martin et al., 1995 ▶). Annexin-V exhibits a high affinityfor PS and recognizes and binds the cell surface with exposed PS. Use of Annexin-V in combination with PI allowed the distinction of early apoptotic and necrotic cells from viable cells. 

Extrinsic and intrinsic pathways are two major apoptotic pathways. The extrinsic is receptor binding, followed by activation of the initiator caspase (*caspase-8*), which in turn leads to activating of *caspase-3* or amplifies *caspase-3* activation by cleaving the *Bcl-2* family (Van Poppel and Tombal, 2011 ▶) The intrinsic or mitochondrial pathway comprises members of the Bcl-2 family that regulate releasing of cytochrome c from the mitochondria. In both pathways, the late apoptotic events occur after activation of *caspase-3 *(Sundquist et al., 2006 ▶). Mitochondrial dysfunction leads to breakdown of mitochondrial permeability transition pore, then cytochrome C releases from mitochondria into cytosol (Chan et al., 2013 ▶). A pro-apoptotic member of the *Bcl2 *family called *BAX*, can induce release of cytochrome (Chan et al., 2013 ▶) from mitochondria into the cytosol, whereas *Bcl2*, an anti-apoptotic member of the *Bcl2* family, can prevent apoptosis to block cytochrome c release from mitochondria. Interestingly, the effects of apoptosis induction are more relevant to the balance between *Bcl-2* and *BAX* than the quantity of Bcl-2 alone. Thus, the *BAX/Bc1-2* ratio is critical for cell survival or death (Yu et al., 2011 ▶) Imbalances of the *BAX/Bc1-2* ratio contributed to induction of apoptosis by mitochondrial pathway (Cory and Adams, 2002 ▶). In this study, *C. chinensis *extract led to disruption of BAX/Bc1-2 ratio, by increasing *BAX* and decreasing *Bcl-2*. These data exhibited that *C. chinensis* induced apoptosis in PC3 and MCF7 cells through mitochondrial-dependent pathway. 

Many chemotherapeutic agents activate apoptosis through caspase-dependent pathway (Shu et al., 2009 ▶). Activation of caspase-3 has a vital role in apoptosis process and suppression of cancer. After cleavage, activated caspase-3 triggers apoptosis by cleaving its substrate PAR leading to DNA fragmentation (Sun et al., 1999 ▶). 

*PTEN* acts as a tumor suppressor gene which is the most mutated or deleted gene located in the cytoplasm and is responsible for lipid and protein dephosphorylation (Cory and Adams, 2002 ▶). It plays a pivotal role in the suppression of tumor cell proliferation, and cell migration, and induction of apoptosis (Li et al., 2007 ▶). Therefore, we expect that *PTEN* expression is more likely to increase in the existence of chemopreventive agents. The results of present study revealed that *C. chinensis* significantly decreased the expression of *Bcl2* and increased the expression of *BAX*, caspase and *PTEN*. 

In conclusion, our results indicate that *C. chinensis* extract has cytotoxic activity in cancerous cells (PC3 and MCF7). The apoptosis leads to the upregulation of *BAX/Bcl2*, *CASPASE3* and *PTEN*, and activation of lactate dehydrogenase. Therefore, *C. chinensis *extract seems to be a good candidate as an anti-tumor agent against prostate and breast cancers. Further studies are necessary to investigate the effective molecular mechanisms of *C. chinensis *extract. 

## Conflicts of interest

The authors have declared that there is no conflict of interest.
